# Comparative Analysis of Nano-Bactericides and Thiodiazole–Copper on Tomato Rhizosphere Microbiome

**DOI:** 10.3390/microorganisms13061327

**Published:** 2025-06-07

**Authors:** Weimin Ning, Xiangwen Luo, Yu Zhang, Shijun Li, Xiao Yang, Xin Wang, Yueyue Chen, Yashuang Xu, Deyong Zhang, Songbai Zhang, Yong Liu

**Affiliations:** 1 Agricultural Science College, Xichang University, Xichang 615000, China; ningweimin@hnu.edu.cn; 2 Key Laboratory of Pest Management of Horticultural Crop of Hunan Province, Hunan Academy of Agricultural Science, Changsha 410125, China; luoxwxihe@hunaas.cn (X.L.); zhang6_30@163.com (Y.Z.); zhangdeyong@hhrrc.ac.cn (D.Z.); 3 Longping Branch, Biology College, Hunan University, Changsha 410125, China; wangxin221@hnu.edu.cn; 4 Yuelushan Laboratory, Changsha 410082, China; 5 College of Plant Protection, Hunan Agricultural University, Changsha 410128, China; wolsjas0205@163.com (S.L.); yangxiao1923@163.com (X.Y.); chenyueyue0203@163.com (Y.C.); 8774006940@163.com (Y.X.)

**Keywords:** tomato, nanoparticles, nano-bactericide, rhizosphere, microbial community

## Abstract

Vegetable crops such as tomato are highly susceptible to various pathogens. Nanoparticles (NPs) are emerging as effective nano-bactericides for managing plant pathogens. Communities of rhizosphere bacteria are essential for plant physiological health and also serve as a critical factor in evaluating the environmental compatibility of NPs. We evaluated the effects of a nano-bactericide (Cu-Ag nanoparticles) and a commercial bactericide (thiodiazole–copper) on the rhizosphere microbiome of tomato. The results show that low and high doses of the two bactericides induced alterations in the bacterial community structure to differing extents. Cu-Ag nanoparticles increased the relative abundance of potentially beneficial bacteria, including *Bacteroidota*, *Gemmatimonadota*, *Acidobacteriota*, and *Actinobacteriota.* Functional prediction revealed that Cu-Ag nanoparticles may affect the metabolic pathways of tomato root rhizosphere microorganisms and regulate the lacI/galR family, which controls virulence factors and bacterial metabolism. This study provides insight into the influence of metal nanoparticles on plant rhizosphere microbiomes and may lay a foundation for the application of nano-bactericides for the environmentally friendly control of plant diseases.

## 1. Introduction

Tomato (*Solanum lycopersicum* L.) is extensively cultivated in temperate, tropical, and subtropical areas [1]. The Food and Agriculture Organization (FAO, 2019) reported that the global production of fresh tomatoes reached nearly 180 million tonnes, cultivated across 4.76 million hectares in 168 countries [2,3]. The tomato plant is also used as a model plant for studying fruit development because it has fleshy fruit, a sympodial stalk, and compound leaf properties that are not found in the rice and Arabidopsis model plants [4,5,6]. However, biotic and abiotic stresses, such as drought, salinity, heat, and pathogenic factors, adversely affect the growth and productivity of tomato crops. The tomato plant is susceptible to over 200 diseases caused by viruses, viroids, bacteria, fungi, oomycetes, and other various pathogens during its cultivation [7,8,9,10,11]. Conventional breeding methods and approaches based on synthetic pesticides have been employed to address these pathogens, but these methods have not achieved complete success, particularly as the extended and persistent application of pesticides has led to the emergence of super-phytopathogens, non-target bioaccumulation, and associated toxicity. More importantly, the continuous application of pesticides has a considerable influence on the nutritional content of tomatoes and the texture and productivity of the soil [12,13,14,15].

The presence of diverse microbial species on a plant is crucial for the host’s life cycle. The rhizosphere is the narrow zone surrounding the root surface and is regarded as one of the most intricate ecosystems, consisting of a variety of different bacteria, fungi, algae, viruses, and protozoa [16,17,18,19]. Rhizosphere microbiomes play a crucial role in promoting plant growth by supplying essential nutrients [20], improving nutrient cycling [21], and increasing soil fertility [22]. More importantly, rhizobacteria can protect the plant from a variety of physiochemical stressors and phytopathogen infections by generating phytohormones [23,24], promoting nitrogen fixation [25], and accelerating phosphate solubilization [26]. Plant growth-promoting rhizobacteria have been utilized as biocontrol agents and growth enhancers in a range of agricultural settings [26,27,28,29]. However, the rhizosphere microbiome is sensitive to minor alterations in biotic and abiotic stresses [30,31].

The widespread application of pesticides presents certain drawbacks, including soil contamination and harm to indigenous species [32]. Due to their distinctive characteristics, nanomaterials have emerged as potentially effective alternatives to traditional agricultural pesticides for managing soil-borne illnesses via various strategies; previously studied nanomaterials include MgONPs, ZnONPs, AgNPs, AuNPs, CuONPs, ChNPs, and FeO [33]. A multitude of studies have indicated that nanoparticles can improve nutrient utilization efficiency, decrease the spread of pathogens, and strengthen the plant’s defense responses to multiple crop pathogens [34,35]. Liang et al. [36] demonstrated that a ZnO nano-pesticide significantly diminished the severity of tomato bacterial wilt disease when compared to a commercial berberine aqueous solution, and had no adverse effects on plant growth. Abd-Sawsan et al. [37] discovered that chitosan nanoparticles displayed antifungal properties against *Fusarium solani—*a pathogen that causes root rot in tomatoes*—*in sandy clay loam, and also promoted the expression of *t*ranscription factors and defense-related genes. Mahmoud et al. [38] revealed that GnFe nanoparticles are effective in eradicating root-knot nematodes and enhancing the tomato yield in soil. However, the stability of nanoparticles in soil, particularly their morphology, may be influenced by soil solution chemistry and microorganisms; as the physicochemical conditions of the soil change, sustaining the colloidal systems of nanoparticles becomes especially challenging [39,40]. Existing research on nanoparticles in disease management has emphasized their anti-disease efficacy and antibacterial mechanisms; however, relatively few studies have explored the alterations in the microbial communities of the plant rhizosphere after exposure to nanomaterials [41,42,43].

Bimetallic nanoparticles are formed by integrating two independent metal nanoparticles and have diverse morphologies and structures [44]; compared to their monometallic counterparts, they usually demonstrate enhanced antibacterial, antifungal, antidiabetic, and anticancer properties due to the synergistic interactions between the two different nanoparticles [45]. We utilized antimicrobial peptides and bimetallic nanoparticles anchored to multiwalled carbon nanotubes to synthesize Cu-Ag nanoparticles, which exhibited excellent broad-spectrum antibacterial activities and effectively protected tomato crops from bacterial wilt; their protective effectiveness is roughly comparable to that of commercial bactericides, namely thiodiazole–copper [46]. Thiodiazole–copper, which has a structural similarity to *N*,*N*-methylene-bis(2-amino-1,3,4-thiadiazole), is extensively used as a commercial agricultural antibacterial agent in China and has demonstrated efficacy against numerous plant diseases [47,48]. However, we have only performed a preliminary investigation of their antibacterial properties.

To acquire a deep understanding of the influence of nanoparticles on plants and provide a basis for future applications, a nano-bactericide (Cu-Ag nanoparticles) and a commercial bactericide (thiodiazole–copper) were utilized to explore the rhizosphere microbiome of tomato plants. Microbiome analysis was performed to investigate alterations in microorganisms in the soil around tomato roots after treatment with high and low dosages of the nano-bactericide and commercial bactericide. The results reveal changes in the rhizosphere microbiome of the tomato plant after the application of nanoparticles and provide a basis for the sustainable development of nano-bactericides in agricultural crops.

## 2. Materials and Methods

### 2.1. Materials

Tomato seeds (cv. zuanhongmeina) were stored at the Key Laboratory of Pest Management of Horticultural Crop of Hunan Province, Hunan Academy of Agricultural Science. The nano-bactericide (Cu-Ag nanoparticles) was synthesized based on our previous experimental study [46]; the commercialized bactericide (thiodiazole–copper) was purchased from Longwan Chemicals Co., Ltd., Zhejiang, China; The MagPure Soil DNA LQ Kit was purchased from Magen Biotechnology Co., Ltd., Guangzhou, China; the Qubit dsDNA Assay Kit was purchased from Life Technologies Pvt Ltd., Carlsbad, CA, USA; Tks Gflex DNA Polymerase was purchased from Takara Biomedical Technology Co., Ltd., Beijing, China; and the DNeasy PowerSoil kit was purchased from Qiagen N.V Co., Ltd., Hilden, Germany.

### 2.2. Tomato Seedling Growth

Surface sterilization of tomato seeds was conducted using a 5% sodium hypochlorite solution for 10 min, followed by three washes with distilled water. Sterilized tomato seeds were placed in small Petri dishes and allowed to germinate on moist filter paper at 28 °C for 72 h. After 7 days of germinating, they were planted in plastic containers and then put in a greenhouse with a light–dark photoperiod of 16:8 h, a temperature of 27 ± 2 °C, and a humidity of 60%. Tomato seeds continuously germinated and developed into seedlings with 3–4 leaves. Seedlings of comparable size were chosen and subsequently put into pots. Seedlings were left to continue growing in the greenhouse.

### 2.3. Tomato Treatment

Consistent experimental greenhouse conditions were maintained for the treatments in accordance with a randomized block design. Thiodiazole–copper was diluted 500-fold and 300-fold according to the instructions, and Cu-Ag nanoparticles were diluted to 20 μL/mL and 30 μL/mL [46] for the following experiment. Tomato plants exhibiting uniform growth were randomly divided into five groups: control tomato (only sterile water was applied) (Group S1), 20 μL/mL Cu-Ag nanoparticles (Group S2), 30 μL/mL Cu-Ag nanoparticles (Group S3), thiodiazole–copper diluted 500-fold (Group S4), and thiodiazole–copper diluted 330-fold (Group S5). For each treatment group, 50 mL of the corresponding treatment solutions were applied directly to the soil for each plant. The control tomato received 50 mL of water per plant and no other solutions. The first treatment began 30 days after the transplantation of the tomato plants. The second and third treatments were administered on days 45 and 60, respectively. To ensure normal growth, besides the above experimental treatments, all tomato plants were grown in the same greenhouse environment, and the timing and volume of irrigation were precisely controlled to be consistent.

### 2.4. Rhizosphere Soil Sample Collection

The roots were detached using sterile tweezers and scissors and placed into sterile 50 mL tubes containing 35 mL of sterile PBS buffer. The tubes were then subjected to agitation on a thermostatic oscillator (Eppendorf AG Co., Ltd., Hamburg, Germany) for 25 min at 28 °C. Following a 20 min wait at room temperature, the roots were removed using sterile tweezers. Centrifugation was then performed at 4 °C, 6000× *g*, for 5 min to remove the supernatant. The rhizosphere soil was immediately stored at 4 °C until DNA extraction.

### 2.5. DNA Extraction and Amplification

DNA was extracted from 2 g of each soil sample using the MagPure Soil DNA LQ Kit (Magen Biotechnology Co., Ltd., Guangzhou, China). Afterward, the quality of the DNA was visually checked by subjecting the samples to 1% agarose gel electrophoresis, and concentrations were measured using a Nano Drop-2000 spectrophotometer (Thermo Fisher Scientific Co., Ltd., Waltham, MA, USA). The DNA served as a template for PCR amplification, conducted using Tks Gflex DNA Polymerase (Takara Biomedical Technology Co., Ltd., Beijing, China) and the universal primers 343 F and 798 R (343F: 5′-TACGGRAGGCAGCAG-3′; 798R: 5′-AGGGTATCTAATCCT-3′), which are the V3-V4 hypervariable sections of the bacterial 16S rRNA gene [49]. The PCR reaction mixture was prepared using 15 μL of 2×Gflex PCR Buffer, 0.5 μL of plant DNA, 1 μL of forward and reverse primers, 1 μL of template DNA, and 0.6 μL of Tks Gflex DNA Polymerase, followed by the addition of double-distilled water to reach a total volume of 30 μL. The following procedures were used to carry out amplification on a PCR thermocycler: DNA predenaturation at 95 °C for 5 min, followed by 26 cycles of denaturation at 94 °C for 30 s, annealing at 56 °C for 30 s, and extension at 72 °C for 20 s, and a single extension at 72 °C for 5 min. The PCR amplicon was purified using AMPure XP beads (Beckman Coulter Co., Ltd., Brea, CA, USA) and quantified with the Qubit dsDNA assay kit (Life Technologies Pvt Ltd., Carlsbad, CA, USA). Equal amounts of the purified amplicon were pooled for subsequent sequencing using the Illumina NovaSeq 6000 platform (Illumina Co., Ltd., San Diego, CA, USA).

### 2.6. Bioinformatic Analysis

The sequencing data of the 16S rRNA gene were saved in fastq format. The fastq files for each sample underwent filtering, trimming, and dereplication to determine the error rates through “DADA2” and QIIME2 (September 2023) [50]. The Chao1 and Shannon indices were calculated using the ASV table in the Q11ME2 platform [51,52]. Beta diversity was analyzed using a principal coordinate analysis (PCoA) and non-metric multidimensional scaling (NMDS) based on the Bray–Curtis distance [53]. The bacterial communities were analyzed at the class, phylum, and genus levels using the “ggplot2” based R package (version 3.5.1) [54]. Biomarkers in differently treated root soils were identified using the linear discriminant analysis (LDA) effect size (LEfSe), with the default LDA score defined above 3.5 [55]. The co-occurrence patterns of the major bacterial community species were assessed to show species correlations utilizing a Spearman correlation matrix; species with Spearman coefficients greater than 0.8 and *p*-values less than 0.05 are shown [56]. Phylo-genetic Investigation of Communities by Reconstruction of Unobserved States (PICRUSt2) (2.3.0b0) software was used to predict the functional composition based on the taxonomy of 16S rRNA gene sequences so that differences in function between different treatment samples could be identified [57]. The experimental data were statistically analyzed by performing one-way analysis of variance (ANOVA) using Graph Pad Prism 10.

## 3. Results

### 3.1. Changes in Microbial Members After Treatment with Nano-Bactericide and Bactericide

**Figure 1:**
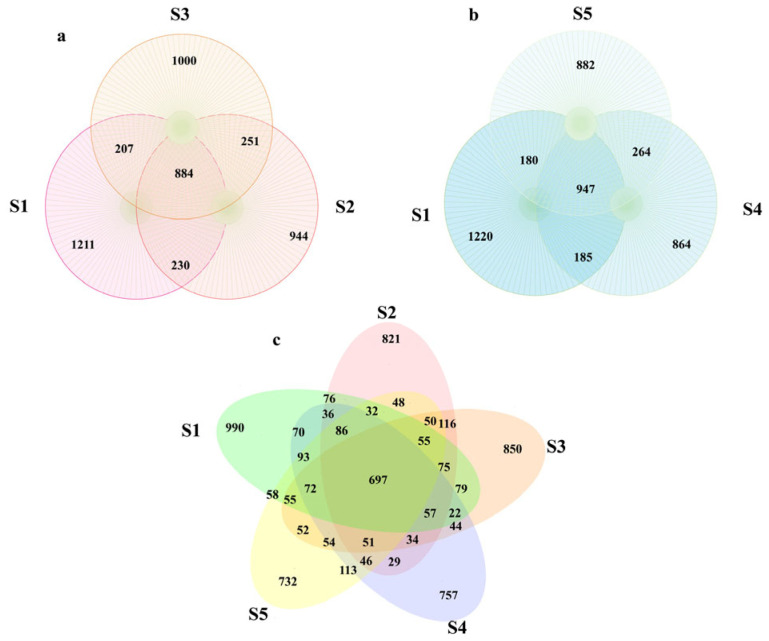
Unique ASVs in Cu-Ag nanoparticles treated group compared to control tomato ASVs (**a**). Unique ASVs in thiodiazole–copper treated group compared to control tomato ASVs (**b**). Unique ASVs between different concentrations of thiodiazole–copper treated group, nanomaterials group and control tomatoes ASVs (**c**). Abbreviations: Tomato plants only treated with the water (S1). Tomato treated with low dosages of Cu-Ag nanoparticles (S2). Tomato treated with high dosages of Cu-Ag nanoparticles (S3). Tomato treated with low dosages of thiodiazole–copper (S4). Tomato treated with high dosages of thiodiazole–copper (S5).

Transmission electron microscopy (TEM) is an effective technique for analyzing the minuscule crystalline internal structure and shape of nanoparticles via electron diffraction. The typical morphology of Cu-Ag nanoparticles is spherical, as shown by micrographs that reveal a narrow size distribution and smooth surfaces (Figure S1). The tomato plants were photographed and measured before sampling. No significant differences were observed in the height of tomato plants between the treatment and control groups, showing that the application of nanomaterials did not influence tomato height (Figure S2). High-throughput sequence analyses were conducted to assess the effects of thiodiazole–copper and Cu-Ag nanoparticle application on tomato rhizosphere microbial communities. Following the removal of chloroplast DNA, mitochondrial DNA, and other non-bacterial sequences, along with conducting quality control, a total of 57,139–64,372 clean tags were isolated. Then, chimeras were removed from the tags, resulting in 53,373–61,838 valid tags, which were subsequently utilized for analysis. These sequences were assigned to a range of 981–1185 amplicon sequence variants (ASVs). The resulting ASVs were subjected to comprehensive analysis to ascertain treatment-specific responses to exogenous material in the tomato rhizosphere. Exposure to varying concentrations of Cu-Ag nanoparticles and thiodiazole–copper impacted the number of bacterial ASVs in the tomato rhizosphere. A total of 2532, 2309, 2342, 2260, and 2273 ASVs were observed in groups S1, S2, S3, S4, and S5, respectively. More ASVs were discovered in the Cu-Ag nanoparticle treatment than in the thiodiazole–copper group. An examination of samples treated with low and high dosages of Cu-Ag nanoparticles and control samples revealed a total of 884 unique ASVs. A comparison of the thiodiazole–copper and control tomato sample datasets revealed that 947 ASVs were shared. Of the total microbial ASVs, 697 were shared by all five treatments (Figure 1).

### 3.2. Microbial Alpha Diversity and Beta Diversity

**Figure 2:**
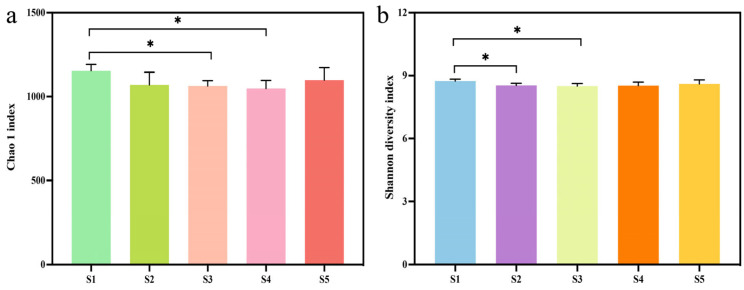
Chao1 (**a**) and Shannon (**b**) index of tomato under the exposure of low and high dosages of thiodiazole–copper and Cu-Ag nanoparticles nanomaterials. Error bars showing the mean of four replicates. Statistical significance was determined based on Tukey’s HSD test. * *p* < 0.05.

**Figure 3:**
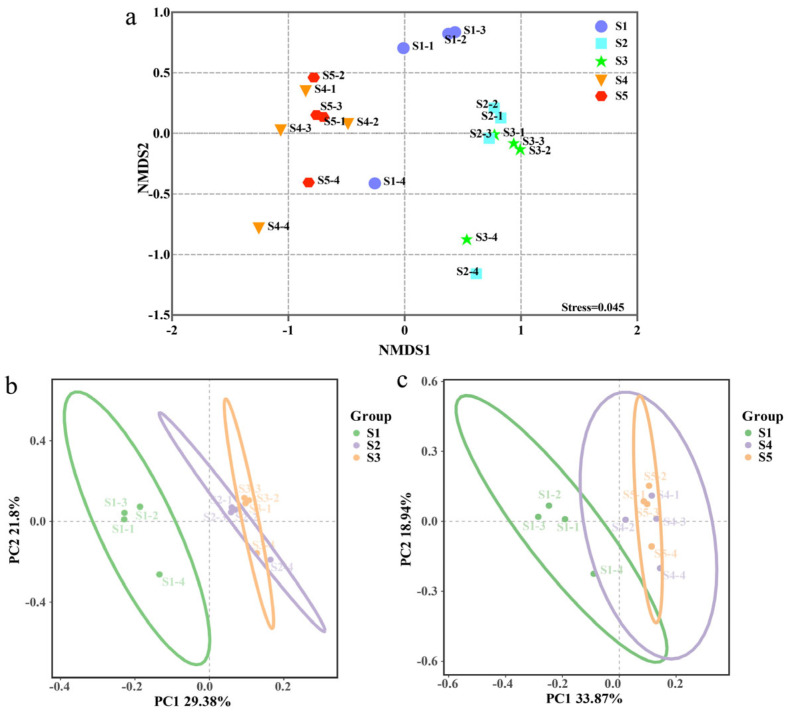
Community clustering of bacteria under different treatments was visualized in a two-dimensional NMDS plot based on a Bray–Curtis matrix (**a**). Principal coordinate analysis (PCoA) based on the Bray–Curtis index dissimilarity of tomato treated with Cu-Ag nanoparticles (**b**). PCoA analysis of tomato samples and tomato treated thiodiazole–copper (**c**).

The Chao1 and Shannon indices were used to evaluate bacterial alpha diversity in each treatment of tomato samples. The Chao1 index demonstrates that a high dosage of Cu-Ag nanoparticles and a low dosage of thiodiazole–copper influenced the rhizosphere diversity of tomato. Meanwhile, the Shannon index shows that the dosage of Cu-Ag nanoparticles had an impact on bacterial diversity in the tomato rhizosphere (Figure 2). To better understand the impact of different treatments on the microbial community, a two-dimensional NMDS plot and PCoA were used. The NMDS plot shows that tomato plants treated with Cu-Ag nanoparticles tended to cluster together, and those treated with a low dosage of thiodiazole–copper also showed clustering behavior alongside a high dosage of thiodiazole–copper treatment (Figure 3a). The PCoA showed that tomato samples treated with both dosages of Cu-Ag nanoparticles and the control plants were distinctly separated along the first axis. The first axis accounts for 29.38% of the overall variation, while the second axis explains 21.8%. Moreover, the first and second axes account for 33.87% and 18.94%, respectively, of the bacterial community alterations between thiodiazole–copper-treated and untreated tomato plants (Figure 3b,c). Overall, tomato plants treated with Cu-Ag nanoparticles and thiodiazole–copper differed in microbial community diversity in the rhizosphere bacterial community.

### 3.3. Tomato Bacterial Composition Affected by Nano-Bactericide and Bactericide

**Figure 4:**
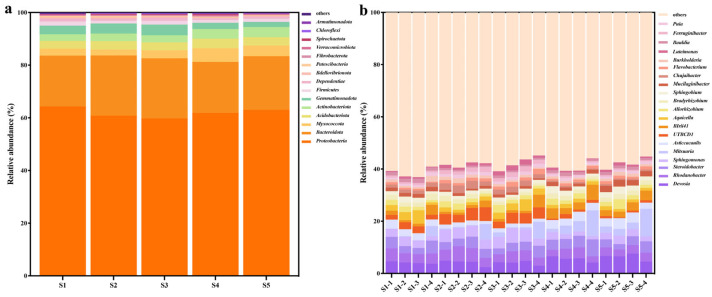
Effect of thiodiazole–copper and Cu-Ag nanoparticles on the bacterial community abundance of tomato. Relative abundance distribution of the top 15 microbial phyla (**a**) and the top 20 bacterial genera (**b**) in the rhizosphere of all tomato samples.

The number of tomato rhizosphere bacteria categorized at the phylum, class, order, family, genus, and species annotation were different between treatments, indicating that Cu-Ag nanoparticles and thiodiazole–copper altered the community structure (Figure S3). The relative variations in the abundance of dominant species in rhizosphere microbial communities were investigated at the phylum, order, and genus levels. First, the bacterial community was investigated at the phylum level. The analysis indicated that *Proteobacteria* was the most abundant phylum of microorganisms in all five groups. The amendment of Cu-Ag nanoparticles resulted in increased relative abundances of *Bacteroidota*, *Gemmatimonadota*, *Acidobacteriota*, and *Actinobacteriota*. Notably, the high dosage of nanomaterials drastically increased the relative abundance of *Gemmatimonadota* when compared to both the control and the lower dosage of nanomaterials. It is fascinating that *Acidobacteriota* and *Actinobacteriota* were the most abundant in the group with the low dosage of nanomaterials. The number of *Myxococcota*, *Firmicutes*, and *Dependentiae* was diminished in soil treated with a low concentration of Cu-Ag nanoparticles. Following the inoculation with thiodiazole–copper, there was a notable increase in the relative abundances of *Myxococcota*, *Actinobacteriota*, and *Acidobacteriota* when compared to the untreated tomato. Nevertheless, the relative abundance of these bacteria decreased as the concentration of thiodiazole–copper increased. Simultaneously, there were no observed differences in *Bacteroidota* following the treatment with thiodiazole–copper. However, the relative abundances of *Gemmatimonadota*, *Firmicutes*, and *Dependentiae* in the thiodiazole–copper treatment group were significantly lower than in the control group (Figure 4a).

The bacterial community at the class level was primarily composed of *Alphaproteobacteria, Gammaproteobacteria*, and *Bacteroidia*, followed by representatives from *Polyangia*, *Acidobacteriae, Gemmatimonadetes*, and *Actinobacteria*. The cumulative percentages of these bacteria were 93.60%, 94.73%, 94.02%, 94.73%, and 95.13% in the S1, S2, S3, S4, and S5 groups, respectively (Figure S4).

In the tomato soil rhizosphere, the predominant bacterial genus among the top 20 was *Devosia*, which was the most abundant in all tomato samples. The relative abundances of *Sphingomonas*, *Mitsuaria*, *UTBCD1*, *Allorhizobium*, *Chujaibacter*, *Flavobacterium*, *Puia*, *Ferruginibacter*, and *Luteimonas* increased after the application of low and high doses of Cu-Ag nanoparticles compared to the control tomato. Both low and high concentrations of Cu-Ag nanoparticles reduced the number of *Devosia*, *Steroidobacter*, *Asticcacaulis*, *Bradyrhizobium*, *Sphingobium*, *Mucilaginibacter*, *Burkholderia*, and *Bauldia.* The low concentration of nanomaterials increased the abundance of *Rhodanobacter*, but the high concentration reduced it; conversely, the low dose reduced the abundances of *Birii41* and *Aquicella*, whereas the high concentration increased them.

The influence of thiodiazole–copper on other bacteria at the genus level were differed. The application of both low and high dosages of thiodiazole–copper caused a decrease in the relative abundances of *Rhodanobacter*, *Sphingomonas*, *UTBCD1*, *Aquicella*, *Bradyrhizobium*, *Chujaibacte*r, *Ferruginibacter*, and *Bauldia*, while they increased the relative abundances of *Devosia*, *Mitsuaria*, *Asticcacaulis*, *BIrii41*, *Allorhizobium*, *Sphingobium*, *Flavobacterium*, and *Luteimonas* (Figure 4b, Table S1). The alterations in the tomato rhizosphere community differed between treatments with Cu-Ag nanoparticles and thiodiazole–copper, showing that the use of nanomaterials could influence microbial structure.

### 3.4. LEfSe Analysis of the Rhizosphere Bacteria

**Figure 5:**
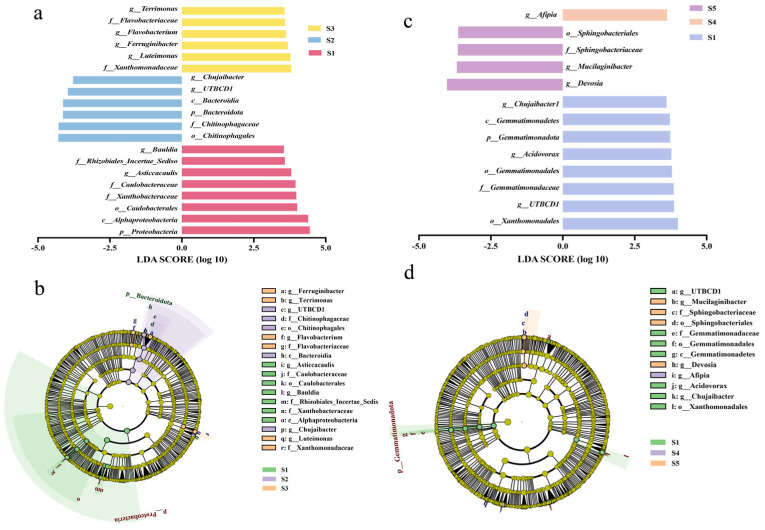
Linear discriminant analysis effect size (LEfSe) of bacteria. Significantly different abundant taxa of tomato rhizosphere from Cu-Ag nanoparticles treatments (**a**,**b**) and thiodiazole–copper treatments (**c**,**d**). The dimensions of the colored dots correspond to the relative abundance of the bacteria. The figure presents species that exhibit notable differences. Abbreviations: c: class; o: order; f: family; g: genus; s: species.

The linear discriminant analysis effect size (LEfSe) can identify biomarkers with significant variations. Linear discrimination analysis (LDA) scores >3.5 were used to reveal the biomarkers with the largest differences in tomato rhizosphere soil microbial communities under different treatment conditions. Alterations in bacterial taxonomic abundance in soil were induced by both low and high concentrations of the nano-bactericide. Tomato plants treated with a low dose of the nano-bactericide were enriched in the genus *Chujaibacter* (3.77), the genus *UTBCD1* (3.96), the class *Bacteroidia* (4.12), the phylum B*acteroidota* (4.13), the family *Chitinophagaceae* (4.28), and the order *Chitinophagales* (4.29). In the rhizosphere of healthy tomato plants treated with a higher dose of the nano-bactericide, the following kinds of bacteria were found: the genus *Terrimonas* (3.81), the family *Flavobacteriaceae* (3.77), the genus *Flavobacterium* (3.68), the genus *Ferruginibacter* (3.62), the genus *Luteimonas* (3.59), and the family *Xanthomonadaceae* (3.57) (Figure 5a,b). Differential alterations were seen in the microbial communities of tomato rhizosphere soil after bactericide treatment. Only the genus *Afipia* (3.62) was abundant in soils treated with a low level of thiodiazole–copper. Four biomarkers were discovered in soil treated with a high concentration of thiodiazole–copper: the genus *Devosia* (4.03), the genus *Mucilaginibacter* (3.68), the family *Sphingobacteriaceae* (3.65), and the family *Sphingobacteriales* (3.64), all of which displayed significant abundance (Figure 5c,d). The LEfSe analysis indicated variations in microbial community biomarkers between tomatoes treated with the nano-bactericide and those treated with the bactericide.

### 3.5. Co-Occurrence Networks Analysis

**Figure 6:**
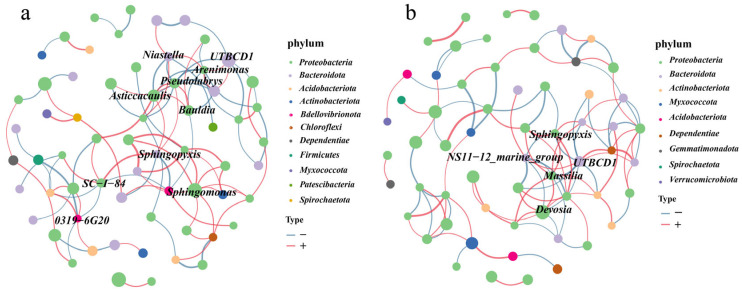
The bacterial community networks in the tomato treated with nano-bactericide (**a**). The community networks in the tomato treated thiodiazole–copper (**b**). The number of nodes, edges, and the clustering coefficients are shown in the networks, and the nodes are colored according to phylum. Pink and blue lines represent negative and positive associations, respectively.

To study the potentially abundant microbiota in tomatoes, the complexity of interactions among tomato microbial communities in different treatments was analyzed using a co-occurrence network. In tomatoes treated with Cu-Ag nanoparticles, the core bacteria *Arenimonas*, *Pseudolabrys*, *Asticcacaulis*, *Sphingopyxis*, *Sphingomonas*, and *SC-1-84* belong to the phylum *Proteobacteria*, whereas *UTBCD1* and *Niastella* belong to the phylum *Bacteroidota*. The bacterium *Pseudolabrys* had the maximum number of connections in the networks and was positively associated with *Asticcacaulis* and *Bradyrhizobium* (Figure 6a). In tomatoes treated with thiodiazole–copper, the core bacteria *Sphingopyxis*, *Massilia*, and *Devosia* belong to the phylum *Proteobacteria*, whereas *UTBCD1 and NS11−12* belong to the phylum *Bacteroidota*. The bacteria *UTBCD1* were densely linked nodes and were negatively associated with *Devosia*, *Massilia*, and *Sphingopyxis* (Figure 6b). The examination of co-occurrence connections revealed differences in the tomato network structure between the nano-bactericide and thiodiazole–copper treatments. The analysis of the node degree distribution indicated a higher number of link nodes in the tomatoes treated with Cu-Ag nanoparticles than in those treated with thiodiazole–copper.

### 3.6. The Functional Prediction of Microbial Communities

PICRUSt2 (2.3.0b0) is a tool to predict functional profiles that can improve the comprehension of microbial community functions in various environments. It places ASVs in a reference tree, serving as the basis for functional predictions, and then generates community-wide pathway abundances. PICRUSt2 functional prediction was performed with the KEGG pathway database to assess possible alterations in bacterial community functions in tomato following the nanomaterial treatments. First, we identified eight metabolic functions that were classified as Level 3 in all tomato samples. The pathways in tomato under various treatment conditions were enriched in metabolic pathways, the biosynthesis of secondary metabolites, microbial metabolism in diverse environments, the biosynthesis of cofactors, the biosynthesis of amino acids, carbon metabolism, ABC transporters, and a two-component system, which all exhibited the highest levels of enrichment in tomato plants treated with a high dosage of thiodiazole–copper and the lowest levels of enrichment in the control tomato. The level of pathway enrichment observed in tomato plants treated with nanomaterials was comparable to that in the control tomato group (Figure S5a, Table S2).

PICRUSt2 predictions were also employed to identify potential changes in enzymes in soil microbial communities. The levels of enzymes corresponding to the ABC-type transport system (K02014) and ATP-binding protein (K01990) showed no notable variations in the bacterial communities of Cu-Ag nanoparticle treatments, irrespective of the dosage. It is noteworthy that the abundances of K02014 and K01990 were reduced by the low dosage of thiodiazole–copper, whereas an increase was observed with the high dosage. More importantly, the expression of virulence factors is regulated by the lacI/galR family transcriptional regulators (K02529), and Cu-Ag nanoparticles and a large dose of thiodiazole–copper enhanced the abundance of the lacI/galR family in the bacterial community (Figure S5b, Table S3). The functional prediction results indicate that Cu-Ag nanoparticles may influence the metabolic pathways of microorganisms in the tomato root rhizosphere.

## 4. Discussion

A broad range of nanomaterials has been used to effectively combat pathogenic microorganisms and address various plant diseases [33]. Previous research has mainly focused on the development of unique nanomaterials and the enhancement of their antibacterial characteristics [38]. Spraying and irrigation are commonly utilized strategies for managing plant diseases with nanomaterials. Both methods have an impact on the plant and the soil, especially the microorganisms inhabiting the soil. Meanwhile, the interactions between microorganisms and plants are essential for promoting plant growth and maintaining health. To investigate the impact of nanomaterials on root rhizosphere microorganisms, the high-throughput sequencing of microorganisms was conducted following tomato root treatment with low and high concentrations of nanomaterials and commercial fungicides.

To better understand the alterations in microbial members in the soil of tomato roots, we analyzed the number of ASVs in tomato rhizosphere microorganisms exposed to various treatments. Fewer ASVs were detected in the thiodiazole–copper group than in the nanomaterial group. Meanwhile, the Chao1 index of tomatoes treated with a low dose of thiodiazole–copper is much lower than that of the control. This may be due to physical and biochemical barriers of the bactericide that restrict microbes from colonizing tomato roots. The nanoparticles contain peptides from a photosynthetic bacterium; these peptides enhance the biocompatibility of the nanoparticles, so soil containing nanoparticles encourages the establishment of microorganisms on the surfaces of roots. The alpha diversity proves that Cu-Ag nanoparticles influence bacterial diversity in the tomato rhizosphere. The NMDS plot indicates that tomato plants treated with either the nano-bactericide or commercial bactericide were distinct from the control tomato. The PCoA analysis showed that the tomato rhizosphere microbial community was divided into three separate groups in the various treatments. Investigations have demonstrated that various environmental factors and seasonal conditions influence bacteria connected to plant hosts [58,59]. These results show that the diversity of tomato rhizosphere bacteria changed with the application of the nano-bactericide and the commercial bactericide.

The rhizosphere community can be influenced by multiple factors, including the host genotype, environment, and soil characteristics [60]. We analyzed the response of tomato rhizosphere bacteria to thiodiazole–copper and Cu-Ag nanoparticles. Diverse microbial community compositions were observed in the treated tomato roots compared to the control tomato. *Proteobacteria* is a broadly dispersed bacterial phylum that plays a crucial role in the transformation of hazardous metals and the physicochemical characteristics of soil. Moreover, they can be substantially affected by soil pH and hazardous metals originating from pesticides [61,62]. The main microbes identified were *Proteobacteria* for both the low and high doses of nanoparticles, suggesting that these concentrations of nanomaterial ions are safe for soil bacteria.

The relative abundances of *Bacteroidota*, *Gemmatimonadota*, *Acidobacteriota*, and *Actinobacteriota* were elevated in the tomato rhizosphere treated with Cu-Ag nanoparticles. Previous research demonstrated that the introduction of earthworms significantly enhanced the yield of *Lilium lancifolium* Thunb., with an increase in the relative abundance of *Actinobacteria* compared to the quicklime-treated group [63]. The rotation of marigold increased the relative abundances of *Actinobacteria* and *Acidobacteria* in soil microbial communities, which could decrease the prevalence of root-knot nematodes in the subsequent tobacco crop [64]. *Bacteroidota* are common in soil ecosystems and perform essential roles in nutrient cycling, host growth, stress resilience, and genomic diversity. Maize treated with organic fertilizers showed an elevated diversity of endophytic bacteria and a high relative abundance of Bacteroidota, which were beneficial for its growth [65], and *Bacteroidota* displayed broad antibacterial activity against various fungal and bacterial plant diseases [66,67]. The quantities of potentially beneficial microbes were increased in tomatoes treated with Cu-Ag nanoparticles. Co-occurrence networks can present a novel viewpoint for analyzing microbial interactions; the bacterial network in the tomato rhizosphere treated with Cu-Ag nanoparticles displayed increased complexity and contained more hub taxa. In conclusion, the utilization of nanoparticles may enhance certain beneficial bacteria in the tomato rhizosphere. Further separation and purification of these microorganisms is required, along with their application with nanomaterials to explore possible synergistic effects.

The KEGG pathway database was employed in PICRUSt2 functional prediction to analyze possible alterations in tomato rhizosphere microbial communities after nanomaterial treatments. The functional prediction results indicate that Cu-Ag nanoparticles may influence the metabolic pathways of microorganisms in the tomato root rhizosphere. ATP-binding metal transporters can influence the survival and growth of certain drug-resistant strains by transporting substrates that alter pathogenesis and virulence. Moreover, ATP-binding proteins were shown to participate in drug efflux, cellular detoxification, and antibiotic activity [68,69,70]. Incubation with different doses of Cu-Ag nanoparticles did not result in a noticeable alteration in the expression levels of tomato ATP-binding proteins. Simultaneously, the predictive results indicate that applying Cu-Ag nanoparticles might enhance the abundance of lacI/galR family transcriptional regulators. The lacI/galR family induces point mutations at crucial amino acid positions to provide functional diversity, and it plays a crucial role in bacterial metabolism and controls the production of hyaluronidase and other virulence factors [71,72]. This finding indicates that nanoparticles may regulate enzymes involved in plant stress tolerance and metabolism. The results were obtained from KEGG predictions; therefore, it is necessary to employ other methodologies, such as real-time PCR, to confirm variations in gene activity and to further investigate the genes involved in the community’s metabolic functionality. Reports demonstrate that plant infection by pathogens may affect the composition and functioning of microorganisms in the root rhizosphere [73]. Future studies could concentrate on determining how nanoparticles attract beneficial microorganisms, help plants adapt to external environmental stress, and increase disease resistance.

## 5. Conclusions

In conclusion, we investigated the effects of varying dosages of a nano-bactericide on the tomato rhizosphere microbial community structure and function. High-throughput sequencing data revealed that Cu-Ag nanoparticles can enrich beneficial microorganisms in *Bacteroidota*, *Gemmatimonadota*, *Acidobacteriota*, and *Actinobacteriota*. Meanwhile, functional predictions indicated that nanoparticles may upregulate enzymes involved in plant stress tolerance and metabolism. This study analyzes the influence of a nano-bactericide on the microbial communities associated with tomato roots, which could provide a theoretical basis for the preventive application of nanoparticles in sustainable agriculture.

## Data Availability

Data availability raw reads have been deposited as a BioProject under accession PRJNA1189425 and BioSample accessions SAMN44960159-SAMN44960178 in the National Center for Biotechnology Information.

## Supplementary Materials

The following supporting information can be downloaded at: https://www.mdpi.com/article/10.3390/microorganisms13061327/s1. Figure S1: Transmission electron microscope images of Cu-Ag nanoparticle, and the scale bar is 100 nm. Figure S2: Pictures of tomato plants from the different treatment groups. The plants were of similar height and showed no visible impairment after the different treatments. (a): control. (b): low dosages of Cu-Ag nanoparticles nanomaterials. (c): high dosages of Cu-Ag nanoparticles nanomaterials. (d): low dosages of thiodiazole-copper. (e): high dosages of thiodiazole-copper. (f): Statistical column graph showing the tomato height under various treatments, ns indicates no significant difference. Figure S3: Taxonomic compositions of the bacterial community abundance at anno level in all tomato. Figure S4: Effect of thiodiazole-copper and Cu-Ag nanoparticles on the tomato bacterial community abundance at the class level. Figure S5: The prediction results for the markedly different metabolic pathways of tomato microbial communities exposed to varying dosages of thiodiazole-copper and Cu-Ag nanoparticles treatment, employing Phylogenetic Investigation of Communities by Reconstruction of Unobserved States version 2 (PICRUSt2) analysis (a). PICRUSt2 predicts the top 15 enzymes in tomato root microbial communities under various conditions, shown as a heatmap graphic (b). Statistics of data were according to the Kruskal–Wallis. Table S1: Percentage (%) distribution of relative abundance of the top 20 bacterial taxa in the rhizosphere of all tomato samples. Table S2: PICRUSt2 predicts the enzymes in tomato microbial communities exposed to varying dosages of thiodiazole-copper and Cu-Ag nanoparticle treatments. Table S3: Functional description based on PICRUSt2 predicted results.
