# Supplementary material for: Comparative Analysis of Nano-Bactericides and Thiodiazole–Copper on Tomato Rhizosphere Microbiome
Source: Microorganisms. 2025 Jun 7;13(6):1327. doi: 10.3390/microorganisms13061327 (PMC12195157; doi:10.3390/microorganisms13061327)
Supplement: Supplementary file 1 [file microorganisms-13-01327-s001.zip › microorganisms-3648960-supplementary.pdf]

Supplementary Materials

Comparative Analysis of Nano-Bactericides and Thiodiazole–Copper on Tomato Rhizosphere Microbiome

Weimin Ning [1,2,3], Xiangwen Luo [2,4], Yu Zhang [2,3,4], Shijun Li [5], Xiao Yang [5], Xin Wang [3], Yueyue Chen [5], Yashuang Xu [5], Deyong Zhang [2,3,4], Songbai Zhang [2,3,4,5,*] and Yong Liu [2,3,4,5,*]

[1] Agricultural Science College, Xichang University, Xichang 615000, China
[2] Key Laboratory of Pest Management of Horticultural Crop of Hunan Province, Hunan Academy of Agricultural Science, Changsha 410125, China
[3] Longping Branch, Biology College, Hunan University, Changsha 410125, China
[4] Yuelushan Laboratory, Changsha 410082, China
[5] College of Plant Protection, Hunan Agricultural University, Changsha 410128, China
* Correspondence: zsongb@hunaas.cn (S.Z.); liuyong@hunaas.cn (Y.L.)

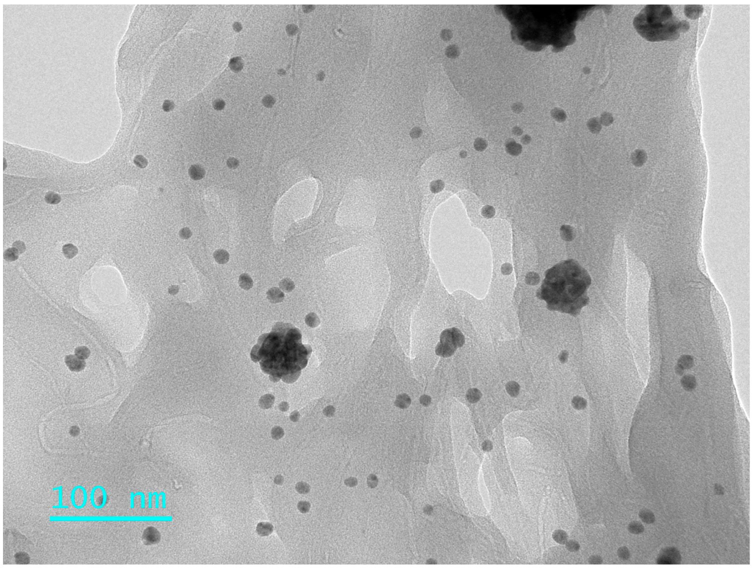

Figure S1. Transmission electron microscope images of Cu-Ag nanoparticle, and the scale bar is 100 nm.

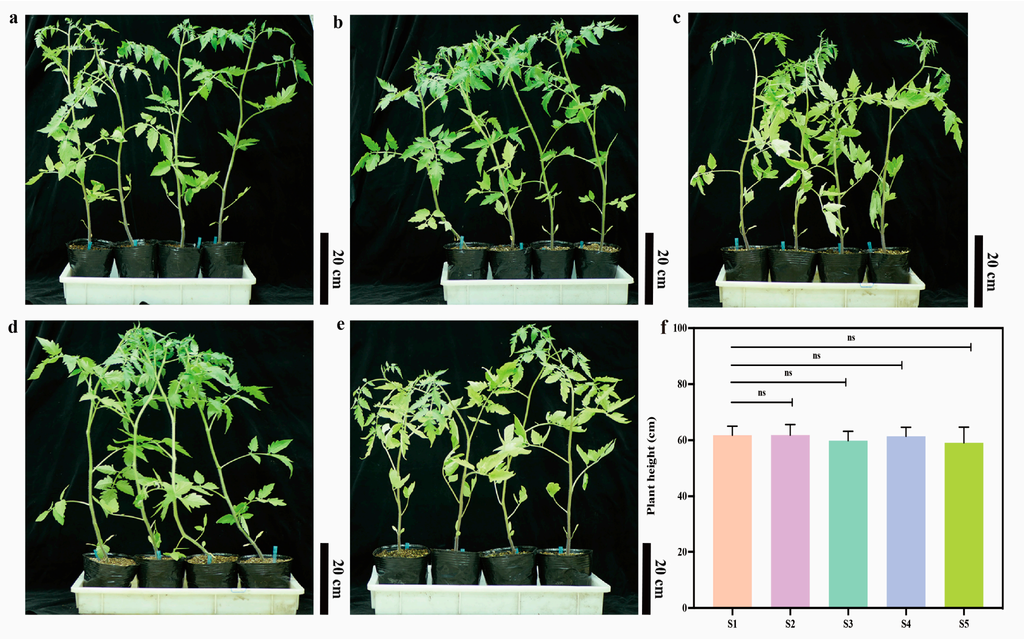

Figure S2. Pictures of tomato plants from the different treatment groups. a: control tomato. b: tomato treated by low dosages of Cu-Ag nanoparticle. c: tomato treated by high dosages of Cu-Ag nanoparticle. d: tomato treated by low dosages of thiodiazole-copper. e: tomato treated by high dosages of thiodiazole-copper. f: Statistical column graph showing the tomato height under various treatments, ns indicates no significant difference.

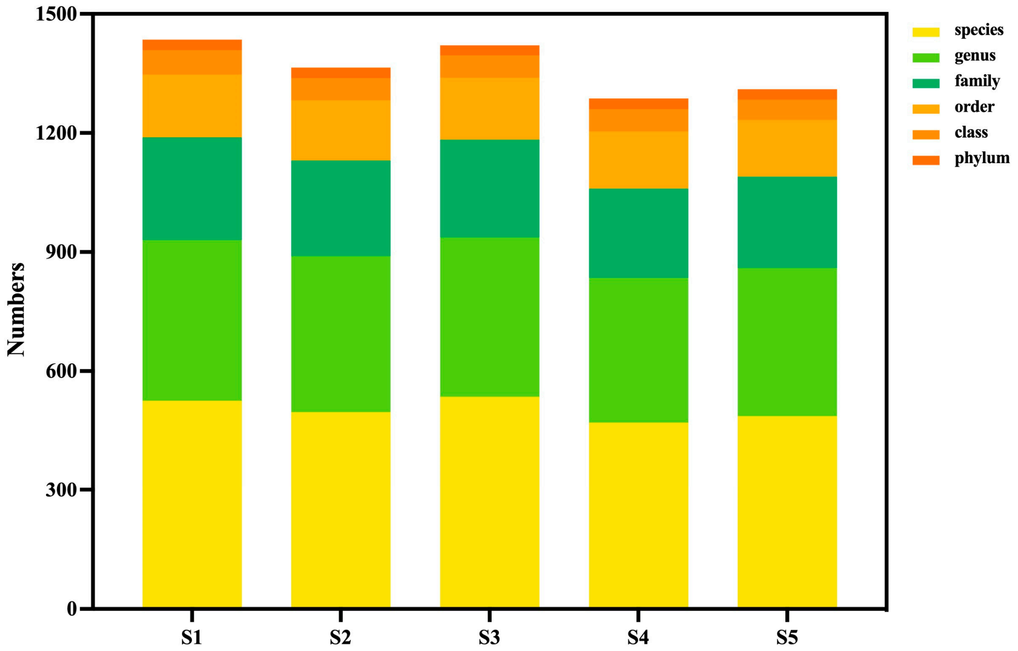

Figure S3. Taxonomic compositions of the bacterial community abundance at annotation level in all tomato.

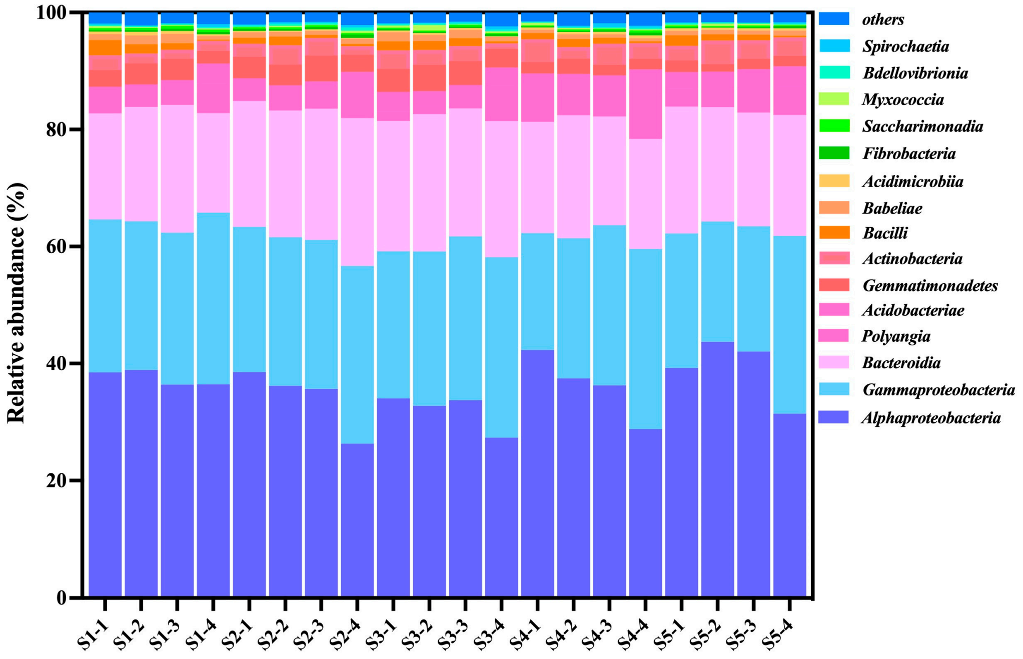

Figure S4. Effect of thiodiazole-copper and Cu-Ag nanoparticle on the tomato bacterial community abundance at the class level.

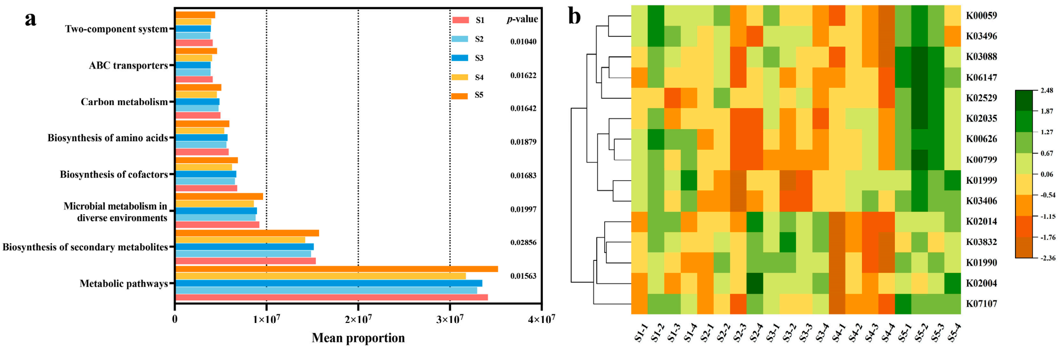

Figure S5. The prediction results for the markedly different metabolic pathways of tomato microbial communities exposed to varying dosages of thiodiazole-copper and Cu-Ag nanoparticles treatment, employing Phylogenetic Investigation of Communities by Reconstruction of Unobserved States version 2 (PICRUSt2) analysis (a). PICRUSt2 predicts the top 15 enzymes in tomato root microbial communities under various conditions, shown as a heatmap graphic (b). Statistics of data were according to the Kruskal-Wallis.

Table S1. Percentage (%) distribution of relative abundance of the top 20 bacterial taxa in the rhizosphere of all tomato samples.

Taxonomy	S1	S2	S3	S4	S5
Devosia	4.119466825	4.08363355	4.05163485	5.5728534	6.332405875
Rhodanobacter	4.00060115	4.70012055	3.64099605	3.18639035	3.185446275
Steroidobacter	3.57639605	3.4238157	3.319188325	4.62681725	3.185345425
Sphingomonas	2.836654925	4.08313865	4.35020485	1.921332675	2.586638475
Mitsuaria	1.025168525	1.79460315	2.31455105	4.584460775	4.51296415
Asticcacaulis	2.854089825	1.579058475	1.4617995	3.155040175	3.394153375
UTBCD1	2.1988759	4.223321125	3.682514025	1.0395641	0.69658305
BIrii41	1.8254983	1.3586412	1.979638625	3.697513525	2.821151575
Aquicella	2.8555124	1.382974825	2.9453239	1.406095375	1.157055575
Allorhizobium	1.5683516	1.768554575	1.739809	1.632987875	2.068879775
Bradyrhizobium	1.85812565	1.635770675	1.1174741	1.620006675	1.78301915
Sphingobium	1.626719725	1.26356145	1.286720575	1.73471355	2.0856554
Mucilaginibacter	1.491133	0.89830935	0.9022379	1.34592645	2.290182925
Chujaibacter	1.34587595	2.516967575	1.92755975	0.528897575	0.58230835
Flavobacterium	0.6392401	1.138486425	1.5524915	0.9319761	1.015034675
Burkholderia	1.149046528	1.355457393	0.82748234	0.754404753	1.136835313
Puia	0.920377903	1.20175651	1.193185618	0.90394735	0.918217345
Ferruginibacter	0.725510853	1.602162125	1.673901658	0.499855658	0.490427298
Bauldia	1.37666701	0.67611358	0.751294905	1.056219973	1.076919533
Luteimonas	0.573115493	1.039810643	1.639942905	0.635312663	0.876950873
others	61.4335723	58.27374248	57.64204858	59.16568375	57.8038256

Table S2. PICRUSt2 predicts the enzymes in tomato microbial communities exposed to varying dosages of thiodiazole-copper and Cu-Ag nanoparticle treatments.

	Two-component system	ABC transporters	Carbon metabolism	Biosynthesis of amino acids	Biosynthesis of cofactors	Microbial metabolism in diverse environments	Biosynthesis of secondary metabolites	Metabolic pathways
S1-1	4028014.98	4092946.93	4862760.57	5724331.64	6625894.41	9009756.66	14988639.82	33302714.69
S1-2	4277493.35	4361123.23	5221064.32	6137278.24	7116667.64	9669583.31	16056765.08	35695142.23
S1-3	3991859.24	3930120.77	4981320.38	5881673.54	6810303.59	9113877.95	15360242.2	33911638.51
S1-4	4242315.02	4139396.09	4874097.45	5757364.97	6663486.99	9092249.97	15072437.68	33639808.27
S2-1	3847764.53	4114331.9	4780912.21	5624181.82	6558428.43	8902176.45	14821761.57	33018499.06
S2-2	3952134.68	4132465.38	4925663.11	5791229.27	6733089.24	9148871.69	15267522.29	33940570.89
S2-3	3605700.92	3628116.22	4605318.11	5440985.92	6329007.13	8452206.71	14334628.77	31645740.16
S2-4	4049003.39	3729491.66	4775441.44	5687755.76	6652170.53	8780091.94	15025598.57	33245695.85
S3-1	4017180.39	4227117.18	5026244.86	5903993.49	6887258.23	9342296.34	15602744.5	34647087.25
S3-2	3822867.89	3727122.19	4879402.02	5747516.91	6706047.29	8879792.33	15164800.61	33371957.88
S3-3	3847169.13	3904939.4	4896412.72	5776950.78	6740991.31	8988075.59	15200075.83	33574890.48
S3-4	4000294.91	3729395.9	4683115.85	5567534.16	6509169.35	8597047.96	14669195.55	32490246.93

Table S2. Cont.

	Two-component system	ABC transporters	Carbon metabolism	Biosynthesis of amino acids	Biosynthesis of cofactors	Microbial metabolism in diverse environments	Biosynthesis of secondary metabolites	Metabolic pathways
S4-2	4024980.07	4146252.46	4748807.24	5586137.69	6460797.5	8891744.88	14708715.85	32787423.83
S4-3	4073704.14	4247621.65	4683537.08	5490992.68	6340636.14	8813344.11	14439279.52	32321882.48
S4-4	3976728.19	3827344.97	4403336.63	5235553.09	6073099.15	8183975.22	13764901.46	30606243.16
S5-1	4284875.03	4522372.13	5119285.44	5984439.46	6918590.09	9603908.33	15784452.15	35225150.58
S5-2	4534572.79	4983031.01	5224725.96	6082467.25	7015740.7	10042253.01	16116693.04	36355563.58
S5-3	4347409.63	4616200.34	5104432.11	5970616	6879731.4	9646365.41	15753905.12	35251153.01
S5-4	4435945.6	4355043.57	4885718.26	5774989.98	6691995.48	9224608.18	15239899.38	34127088.37
pvalue	0.0156338	0.028559716	0.019969417	0.016827933	0.018787063	0.016420351	0.016220177	0.010403123
significant	*	*	*	*	*	*	*	*

Table S3. Functional description based on PICRUSt2 predicted results.

Function	Description
K00059	fabG; 3-oxoacyl-[acyl-carrier protein] reductase [EC:1.1.1.100]
K03496	parA, soj; chromosome partitioning protein
K03088	rpoE; RNA polymerase sigma-70 factor, ECF subfamily
K06147	ABCB-BAC; ATP-binding cassette, subfamily B, bacterial
K02529	lacI, galR; LacI family transcriptional regulator
K02035	ABC.PE.S; peptide/nickel transport system substrate-binding protein
K00626	E2.3.1.9, atoB; acetyl-CoA C-acetyltransferase [EC:2.3.1.9]
K00799	GST, gst; glutathione S-transferase [EC:2.5.1.18]
K01999	livK; branched-chain amino acid transport system substrate-binding protein
K03406	mcp; methyl-accepting chemotaxis protein
K02014	TC.FEV.OM; iron complex outermembrane recepter protei
K03832	tonB; periplasmic protein TonB
K01990	ABC-2.A; ABC-2 type transport system ATP-binding protein

Table S3. Cont.

Function	Description
K02004	ABC.CD.P; putative ABC transport system permease protein
K07107	ybgC; acyl-CoA thioester hydrolase [EC:3.1.2.-]
